# Non-contrast CT findings suggestive of secondary intracerebral haemorrhage

**DOI:** 10.1093/esj/aakaf010

**Published:** 2026-01-01

**Authors:** Umberto Pensato, Costanza M Rapillo, Federico Mazzacane, Giorgio Busto, Jawed Nawabi, Enrico Fainardi, Gregoire Boulouis, Andreas Charidimou, Marco Pasi, Javier M Romero, Alessandro Padovani, Simona Marcheselli, Joshua N Goldstein, Andrew M Demchuk, Andrea Morotti

**Affiliations:** Department of Neurology, IRCCS Humanitas Research Hospital, via Manzoni 56, 20089 Rozzano, Milan, Italy; Department of Biomedical Sciences, Humanitas University, via Rita Levi Montalcini 4, 20072 Pieve Emanuele, Milan, Italy; Department of Neurology, IRCCS Humanitas Research Hospital, via Manzoni 56, 20089 Rozzano, Milan, Italy; Department of Brain and Behavioral Sciences, University of Pavia, Pavia, Italy; Neuroradiology Unit, Department of Radiology, Careggi University Hospital, Florence, Italy; Department of Neuroradiology, Charité—Universitätsmedizin Berlin, Campus Mitte Humboldt-Universität zu Berlin, Freie Universität Berlin, BIH, Berlin, Germany; Neuroradiology Unit, Department of Radiology, Careggi University Hospital, Florence, Italy; Diagnostic and Interventional Neuroradiology, Tours University Hospital Centre d’Investigation Clinique - Innovation Technologique (CIC-IT), and Université de Tours, INSERM, Imaging Brain & Neuropsychiatry iBraiN, U1253, Tours 37032, France; Department of Neurology, Boston University Chobanian & Avedisian School of Medicine, 85 East Concord Street 1st floor, Boston, MA, United States; Neurology Service, VA Boston Healthcare System, Boston, MA, United States; Department of Neurology, Stroke Unit, Tours University Hospital National Institute of Health and Medical Research U1253 iBrain, Tours, Centre-Val de Loire, France; Neurology Service, VA Boston Healthcare System, Boston, MA, United States; Department of Neurology, Stroke Unit, Tours University Hospital National Institute of Health and Medical Research U1253 iBrain, Tours, Centre-Val de Loire, France; Department of Neuroradiology, Massachusetts General Hospital, Boston, MA, United States; Neurology Unit, Department of Clinical and Experimental Sciences, University of Brescia, Brescia, Italy; Department of Neurology, IRCCS Humanitas Research Hospital, via Manzoni 56, 20089 Rozzano, Milan, Italy; Department of Emergency Medicine, Massachusetts General Hospital, Boston, MA, United States; Calgary Stroke Program, Department of Clinical Neurosciences and Radiology, University of Calgary, Calgary, Alberta, Canada; Neurology Unit, Department of Clinical and Experimental Sciences, University of Brescia, Brescia, Italy

**Keywords:** intraparenchymal haemorrhage, aetiologies, secondary causes, differential diagnosis, macrovascular lesions, haemorrhagic stroke, tumours

## Abstract

**Introduction:**

Most patients with intracerebral hemorrhage (ICH) are initially evaluated using non-contrast CT (NCCT) alone, which may delay or miss diagnoses of secondary causes and limit opportunities for timely targeted intervention. This review aims to identify NCCT findings suggestive of secondary ICH aetiologies.

**Methods:**

We conducted a systematic literature review. Studies were included if they reported NCCT findings in patients with secondary ICH. We excluded studies focusing exclusively on traumatic ICH or anticoagulation-related ICH. Non-contrast CT findings suggestive of secondary ICH were broadly categorised into 4 domains: (i) intra-parenchymal haemorrhage findings, (ii) extra-parenchymal haemorrhage findings, (iii) non-haemorrhagic findings and (iv) absence of small vessel disease (SVD) findings.

**Results:**

We identified a range of NCCT findings that mark an increased likelihood of being associated with secondary ICH. Intraparenchymal haemorrhage findings included morphological characteristics or atypical morphologies (eg, “cashew nut sign”, “flame” shape bleeds, calcifications, fluid levels and disproportionate perihaematomal oedema) as well as unusual anatomical locations (eg, multiple bleeds, location outside the deep supratentorial regions, haemorrhages adjacent to typical arterial aneurysmal sites or venous structures). Extra-parenchymal haemorrhage findings included haemorrhage extension into intraventricular, subdural or subarachnoid spaces, and isolated intraventricular haemorrhage. Non-haemorrhagic findings included concomitant ischaemic lesions and venous hyperdensity. The absence of SVD markers also suggested secondary ICH.

**Conclusion:**

Several NCCT findings can raise suspicion for secondary ICH and may guide early decision-making regarding the need for further imaging beyond NCCT. Recognising these findings is especially valuable in settings with limited access to advanced diagnostics.

## Graphical Abstract

**Graphical Abstract:**
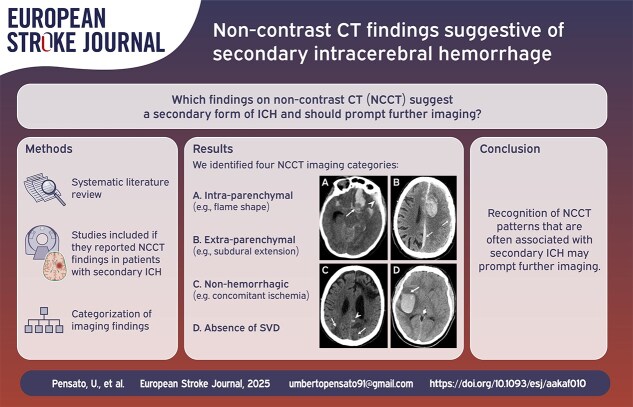

## Introduction

Non-traumatic intracerebral hemorrhage (ICH) is a severe form of stroke caused by the rupture of a blood vessel within the cerebral parenchyma or ventricular system.^1^ Although it accounts for a minority of all acute strokes, it contributes disproportionately to global stroke-related morbidity and mortality, being responsible for nearly half of the global stroke burden.^2^ Most ICH cases are attributed to cerebral small vessel disease (SVD), including arteriolosclerosis (also known as hypertensive arteriopathy) and cerebral amyloid angiopathy.^1^^,^^3^ However, 10%–20% of ICH cases are due to secondary aetiologies, such as macrovascular lesions (eg, arteriovenous malformations [AVMs], intracranial aneurysms, dural arteriovenous fistulas [DAFs] or cavernous malformations), cerebral venous thrombosis (CVT), haemorrhagic transformation of infarction, vasculitis or related vasculopathies, brain tumours (primary or metastatic), haemostatic disorders and other rare entities.^1^^,^^3^^,^^4^

While general management principles apply across ICH aetiologies, secondary causes often require aetiology-specific interventions that may be inappropriate, or even harmful, if applied to presumed SVD-related ICH. For example, anticoagulation is lifesaving for CVT but contraindicated in most other acute intracranial haemorrhagic conditions.^4^^,^^5^ Although brain MRI, CT/MR angiography and digital subtraction angiography are often needed to identify secondary ICH causes, non-contrast CT (NCCT) remains the most widely available and commonly used imaging modality in the acute setting. As a result, diagnoses of secondary ICH may be delayed or entirely missed, limiting opportunities for targeted intervention.^1^

In this study, we systematically review the literature to identify NCCT findings suggestive of a secondary aetiology of ICH, aiming to support early diagnostic stratification and guide decisions about further imaging—particularly in resource-limited settings.

## Patients and methods

### Search strategy

We performed a comprehensive review of the literature to ensure the inclusion of all available evidence on NCCT findings suggestive of secondary ICH. We used the following terms with no language restrictions to search MEDLINE (PubMed), Scopus and EMBASE from inception to 30 April 2025: ((Intracerebral Hemorrhage[Mesh] OR “Intracerebral Hemorrhage” OR “Intracerebral Hematoma” OR “Intracranial Hemorrhage”)) AND (“secondary”[Title/Abstract] OR “etiolog^*^”[Title/Abstract] OR “classification”[Title/Abstract]) AND (Computed Tomography[Mesh] OR CT[Title/Abstract] OR “non-contrast CT”[Title/Abstract] OR “noncontrast CT”[Title/Abstract] OR “NCCT”[Title/Abstract]).

### Screening and selection of studies

We included review articles, randomised controlled trials, observational studies, guidelines, case series and case reports. No language restrictions were applied. Potentially relevant titles and abstracts were imported into Covidence systematic review software, and 2 authors (C.M.R. and F.M.) performed independent screening and full-text review for relevance. A third author (U.P.) resolved disagreements. We selected articles that included patients with secondary ICH who were investigated with NCCT. Articles referring exclusively to traumatic ICH and anticoagulation-related ICH were excluded. The latter were excluded because antithrombotic therapy is now widely regarded as a risk factor for ICH rather than a true underlying aetiology.^3^ In addition to the results of the systematic search, we also included relevant articles identified through manual reference checking, or co-author suggestions.

### NCCT findings categorisation and selection

Non-contrast CT findings suggestive of secondary ICH were broadly categorised into 4 domains, based on the authors’ consensus: (i) intra-parenchymal haemorrhage findings, (ii) extra-parenchymal haemorrhage findings, (iii) non-haemorrhagic findings and (iv) absence of typical SVD findings. This pragmatic categorisation, not derived from any previously published framework, was adopted to facilitate descriptive analysis. Non-contrast CT findings were selected based on evidence-based findings from the scoping review search or expert opinion.

## Results

### Search results and included studies

**Figure 1:**
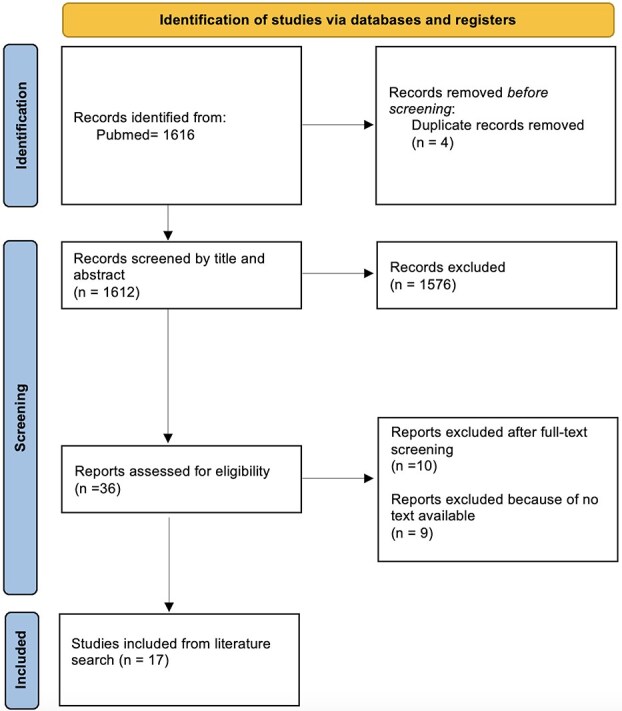
PRISMA flow diagram for study selection. Abbreviation: PRISMA = Preferred Reporting Items for Systematic Reviews and Meta-Analyses.

A total of 1616 studies were screened, of which 4 were removed as duplicates, 1576 were excluded based on title/abstract review, 9 were excluded due to no text available and 7 were excluded after full-text assessment. Overall, 35 studies were included in the scoping review, 17 from the literature search^6–22^ and 16 from the reference list screening or co-authors’ suggestions^3^^,^^4^^,^^23–36^ (Figure 1).

**Figure 2:**
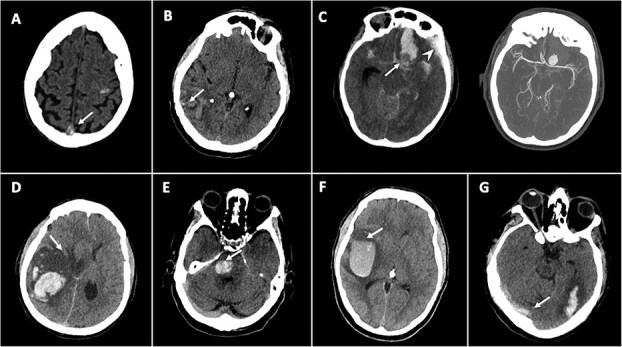
Examples of intra-parenchymal haemorrhagic NCCT findings suggestive of secondary causes of ICH. (A) Small, concave-shaped haemorrhage in the left frontal Rolandic juxtacortical white matter (“cashew nut sign)” with spontaneous hyperdensity of the superior sagittal sinus (arrowhead) suggestive of cerebral venous thrombosis. (B) Right parietal haemorrhage with calcifications (arrow) suggestive of an arteriovenous malformation. (C) NCCT shows a “flame” shape haemorrhage in the left paramedian frontal lobe near a round hypodensity—visible as a concave, “minus” in the haemorrhage (arrow) with associated subdural haemorrhage (arrowhead). Multidetector CTA shows a large saccular aneurysm of the left middle cerebral artery. (D) Large, irregular ICH with a disproportionate perihaematomal oedema (arrow), suggestive of an underlying tumour. (E) Round hyperdense pontine lesion with no surrounding oedema, consistent with cavernoma. (E) Large right temporal haemorrhage with hypodense and hyperdense regions separated by a horizontal line (“fluid-level”) (arrow), consistent with a coagulopathy or haematological disorder. (F) “Flame” shape haemorrhage with spontaneous hyperdensity of right transverse sinus (arrow), suggestive of venous sinus thrombosis. Abbreviation: NCCT = non-contrast CT.

**Figure 3:**
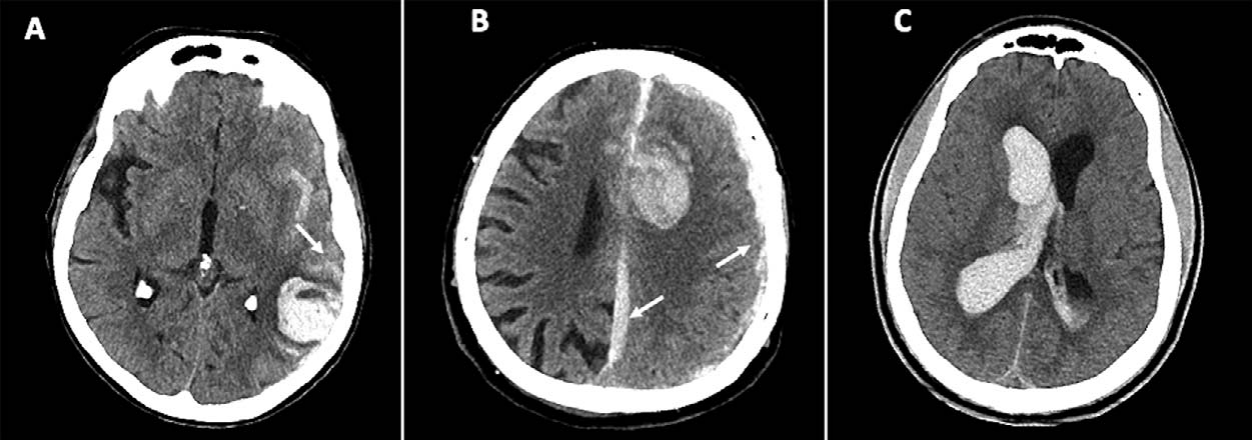
Examples of extra-parenchymal haemorrhagic NCCT findings suggestive of secondary causes of ICH. (A) Haemorrhage in the left temporal lobe with associated subarachnoid haemorrhage in the sulci and the Sylvian fissure (arrow), suggestive of arteriovenous malformation. (B) Haemorrhage in the left frontal lobe with associated subdural haemorrhages of the falx cerebri and the left hemispheric convexity (arrows), suggestive of arteriovenous malformation or cerebral venous thrombosis. (C) Isolated intraventricular haemorrhage suggestive of an arteriovenous malformation. Abbreviation: NCCT = non-contrast CT.

**Figure 4:**
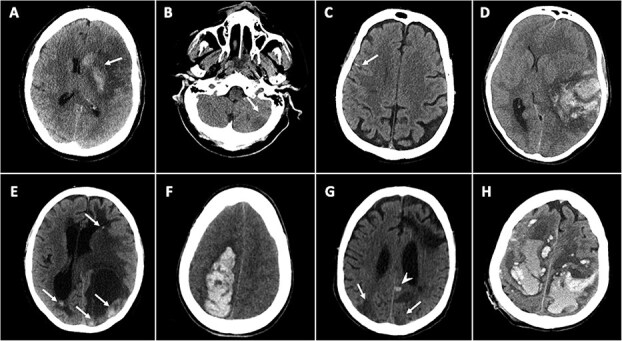
Examples of non-haemorrhagic NCCT findings suggestive of secondary causes of ICH. (A) Left lenticulo-striatal haemorrhage (arrow) in the context of an ischaemic stroke in the superior middle cerebral artery territory, suggestive of haemorrhagic transformation. (B) Small hypodensity in the left cerebellar hemisphere (B) in a non-arterial vascular territory, suggestive of venous infarction. (C) Multiple linear hyperdensities in frontal sulci indicating cortical venous thrombosis. (D) Irregular and heterogeneous haemorrhage with absence of small vessel disease CT markers (no leukoaraiosis or lacunes). (E) Multiple haemorrhages with surrounding oedema secondary to embolic strokes. (F) Right parieto-frontal haemorrhage with absence of adjacent of CT features typically associated with sporadic cerebral amyloid angiopathy (ie, finger-like projections and focal subarachnoid haemorrhages). (G) Multiple, concomitant hypodense and hyperdense lesions (arrows) in a patient with a PRES. (H) Multiple haemorrhages in a patient with acute leukemia, “polka dot sign”. Abbreviations: NCCT = non-contrast CT; PRES = posterior reversible encephalopathy syndrome.

Tables 1 and 2 summarise NCCT findings suggestive of secondary causes of ICH, categorised by imaging features and underlying aetiology. Figures 2–4 show example cases of intra-parenchymal and extra-parenchymal haemorrhagic and non-haemorrhagic NCCT findings that suggest a secondary aetiology.

**Table 1:** NCCT findings suggestive of secondary causes of ICH categorised by imaging features

**Intra-parenchymal haemorrhage NCCT findings**	Morphology	“Cashew nut” sign (CVT) Disproportionate perihaematomal oedema (tumour, CVT) Calcifications (AVM, cavernomas) Fluid-level sign (haematological disorders) Isolated, round, homogenous density and no surrounding oedema (cavernoma) “Flame” shape (AVM, aneurysmal, DAF, CVT) Concave, “minus” shape (aneurysmal)
	Location	Small juxtacortical (CVT) Multiple haemorrhages (CVT, tumour, coagulopathy, vasculitis) Lobar/infratentorial (all secondary causes) Adjacent to venous structures (CVT) Adjacent to arterial aneurysmal rupture sites (aneurysmal) Within arterial ischaemic territory (haemorrhagic transformation) Corpus callosum (primary brain tumour)
**Extra-parenchymal haemorrhage NCCT findings**		Extension to deep subarachnoid or subdural spaces (macrovascular causes, CVT) Isolated intraventricular haemorrhage (AVMs or intraventricular tumours)
**Non-haemorrhagic NCCT findings**		Spontaneous hyperdensity of venous structures, “cord sign” or “triangle sign” (CVT) Ischaemic lesion with arterial territory (haemorrhagic transformation) Ischaemic lesions without arterial territory (CVT) Coexisting haemorrhagic and ischaemic lesions (PRES^*^, RCVS, vasculitis, endocarditis) Hyperdense tubular structures or enlarged vessels (AVM)
**Absence of SVD NCCT findings**		Absence of SVD markers such as white matter hypodensities, old lacunar infarcts and atrophy Absence of amyloid angiopathy signs such as finger-like projections and adjacent focal subarachnoid haemorrhage

Abbreviations: AVM = arteriovenous malformation; CVT = cerebral venous thrombosis; DAF = dural arteriovenous fistula; NCCT = non-contrast CT; PRES = posterior reversible encephalopathy syndrome; RCVS = reversible cerebral vasoconstriction syndrome; SVD = small vessel disease.

Examples of regions adjacent to venous structures include the temporo-lateral region (drained by the vein of Labbe), the parasagittal region (along the superior sagittal sinus) or bilateral thalami (drained by deep cerebral veins). Examples of adjacent areas to typical aneurysmal rupture sites include the temporo-polar region (middle cerebral artery) and paramedian frontal lobe (anterior communicating artery). ^*^In PRES cases, hypodensities usually reflect vasogenic oedema rather than true ischaemia (cytotoxic oedema).

**Table 2:** NCCT findings suggestive of secondary causes of ICH categorised by underlying aetiology

**Arteriovenous malformation**	Location: lobar or infrantentorial; extension to deep subarachnoid or subdural spaces; isolated intraventricular haemorrhage Morphology: multiple calcifications within the haemorrhage; “flame” shape Associated features: hyperdense tubular structure or enlarged vessel
**Aneurysm**	Location: lobar or infratentorial location; adjacent to aneurysmal rupture site Morphology: “flame” shape; concave “minus” shape
**Cavernous malformation**	Location: lobar or infratentorial; extension to deep subrachnoid or subdural spaces Morphology: multiple calcifications within the haemorrhage; isolated, round, homogenous density and no surrounding oedema
**Dural arteriovenous fistula**	Location: lobar or infrantentorial location; extension to deep subarachnoid or subdural spaces Morphology: “flame” shape
**Cerebral venous thrombosis**	Location: multiple; small, juxtacortical haemorrhages; disproportionate oedema; adjacent to venous structures Morphology: “cashew nut” sign, “flame” shape Associated features: spontaneous hyperdensity of venous structures, “cord sign” or “triangle sign”; ischaemic lesions without an arterial territory
**Haemorrhagic transformation of in infarction**	Location: posterior insular region; lobar Associated feature: adjacent ischaemic lesion within an arterial territory
**Tumours (primary or metastatic)**	Location: lobar or infratentorial; multiple; isolated intraventricular haemorrhage; corpus callosum Morphology: disproportionate perihaematomal oedema
**Haematological disorders**	Location: lobar or infratentorial; multiple Morphology: “fluid-level sign”
**Vasculitis and vasculopathies**	Location: multiple; lobar or infrantentorial Associated features: coexisting haemorrhagic and ischaemic/hypodense lesions
**Infectious disorders (systemic infections, CNS infections, endocarditis)**	Location: multiple; lobar or infrantentorial Associated features: coexisting haemorrhagic and ischaemic/hypodense lesions

Abbreviations: CNS = central nervous system; NCCT = non-contrast CT; SVD = small vessel disease.

Examples of regions adjacent to venous structures include the temporo-lateral region (drained by the vein of Labbe), the parasagittal region (along the superior sagittal sinus), or the bilateral medial thalami (drained by deep cerebral veins). Examples of adjacent areas to typical aneurysmal rupture sites include the temporo-polar region (middle cerebral artery) and paramedian frontal lobe (anterior communicating artery).

All secondary causes share the absence of hypertensive and amyloid SVD markers.

### Intra-parenchymal haemorrhage NCCT findings

Several distinct intra-parenchymal NCCT findings, including the morphology and location of the haemorrhage, may suggest specific underlying secondary ICH aetiologies. Small juxtacortical haemorrhages, particularly with a “cashew nut” morphology, were frequently associated with CVT.^23^^,^^24^ This sign is almost pathognomonic of superior sagittal sinus thrombosis, with an observed specificity above 95%.^23^^,^^24^ Disproportionate perihaematomal oedema relative to haematoma volume suggested an underlying neoplastic process or, more rarely, CVT.^3^^,^^6^^,^^12^^,^^15^^,^^16^^,^^22^ One study showed that a relative perihaematomal oedema ratio (perihaematomal oedema volume divided by ICH volume) greater than 0.70 in acute ICH was the optimal threshold for discriminating neoplastic from non-neoplastic haemorrhages.^26^ Calcifications along the margins of or within the haemorrhage have been associated with arteriovenous malformations.^3^^,^^10^^,^^15^^,^^16^^,^^19^^,^^22^^,^^25^ Calcifications with minimal peripheral oedema have also been associated with cavernous malformations.^16^^,^^22^ The presence of a fluid level within the haematoma (“fluid-level sign”) was highly specific for haematological disorders.^15^^,^^16^^,^^22^^,^^28^^,^^29^ Although in most cases it is associated with drug-induced anticoagulation—which is considered a risk factor rather than a direct cause—it might also be associated with intrinsic haematological disorders. A “flame” shape has been associated with AVM, DAF, aneurysm or CVT causes.^35^^,^^36^ A concave, “minus” shape along the border of a haemorrhage may suggest the presence of an intracranial aneurysm, which can be observed as the complementary circle that forms around the area.

The presence of multiple or bilateral haemorrhages might suggest CVT or metastatic brain tumours.^16^ Multifocal small haemorrhages might also be suggestive of vasculitis or haematological disorders,^10^^,^^22^ known also as “polka dot sign.”^27^ Locations other than deep supratentorial were more commonly associated with secondary causes, and infratentorial locations were more so than lobar locations.^6^^,^^21^ Haemorrhages adjacent to venous structures, such as the temporolateral region (drained by the vein of Labbe), parasagittal region (along the superior sagittal sinus) or bilateral medial thalami (drained by deep cerebral veins), should raise suspicion for a venous aetiology.^3^ Similarly, haemorrhage located in typical aneurysmal rupture sites—including the temporo-polar region (middle cerebral artery aneurysm) and paramedian frontal lobe (anterior communicating artery aneurysm)—should raise suspicion of an aneurysmal rupture,^34^ regardless of a positive history for intracranial aneurysm. Haemorrhage involving the corpus callosum suggests a primary brain tumour.^8^

The involvement of an arterial ischaemic territory, in particular with involvement of the cortex, can be suggestive of haemorrhagic transformation.^16^ A haemorrhage with a round-oval shape, homogeneous density and no extension to other brain compartments has been associated with cavernous malformation,^9^^,^^20^ especially when located in the midbrain.^3^

### Extra-parenchymal haemorrhage NCCT findings

Extension of the intraparenchymal haemorrhage into extra-parenchymal compartments may also suggest a secondary ICH aetiology. Extension into the deep subarachnoid or subdural space has been associated with CVT and vascular lesions such as intracranial aneurysms and DAFs.^3^^,^^5^ Isolated intraventricular haemorrhage was often found to be associated with macrovascular causes such as AVMs (up to 25% of cases) or intraventricular tumours.^30^

### Non-haemorrhagic NCCT findings

Non-haemorrhagic findings on NCCT can provide important clues to a secondary cause of ICH. Hyperdensity within venous structures draining the haemorrhage territory, such as the “cord sign” or “triangle sign,” is suggestive of CVT.^16^^,^^19^^,^^25^ This sign offers high specificity yet moderate sensitivity for CVT.^31^^,^^32^ An ischaemic lesion within an arterial territory that is adjacent to a haemorrhage should raise concern for haemorrhagic transformation of an acute infarct.^3^^,^^15^ Conversely, ischaemic lesions that are either remote from the ICH or bilateral (eg, deep venous thalamic infarcts) or do not correspond to a typical arterial distribution are more consistent with venous infarction and may also point towards CVT. The presence of coexisting ischaemic and haemorrhagic lesions has also been associated with other secondary ICH causes, including posterior reversible encephalopathy syndrome (PRES), reversible cerebral vasoconstriction syndrome and vasculitis.^3^ In particular, asymmetrical parieto-occipital oedematous lesions are characteristic of PRES.^4^ Moreover, infectious disorders, including central nervous system infections (eg, herpes simplex virus), systemic infections (eg, sepsis), chronic infection (eg, HIV) and endocarditis, can similarly present with both ischaemic and haemorrhagic findings (Figure S1).^3^^,^^37^^,^^38^ The detection of hyperdense tubular structures/enlarged vessels may indicate the presence of an underlying AVM.^16^^,^^19^^,^^25^

### Absence of SVD NCCT findings

The absence of non-haemorrhagic CT markers of SVD (eg, white matter disease, lacunes and significant atrophy) or, in the case of lobar haemorrhage, the absence of amyloid haemorrhagic signs (eg, finger-like projections and adjacent focal subarachnoid haemorrhage) should also prompt consideration of a secondary cause of ICH.^3^^,^^33^

### Other rare causes of secondary ICH

Other rarer secondary causes of ICH that can be detected on NCCT include remote postsurgical ICH and Duret haemorrhage.^4^ Remote postsurgical ICH is a rare complication of supratentorial craniotomy that usually involves the superior portions of the cerebellum. In contrast, Duret haemorrhages occur in the fourth floor of the ventricles due to damage to the basilar perforators artery by a transtentorial herniation.

## Discussion

We identified a range of NCCT findings that may assist in detecting secondary causes of non-traumatic ICH during the acute phase. These findings were broadly categorised into 4 groups: (i) intraparenchymal haemorrhagic characteristics related to morphology, including atypical morphologies (eg, calcifications, disproportionate perihaematomal oedema and fluid levels) and atypical anatomical locations or patterns (multiple haemorrhages, bleeds adjacent to typical arterial aneurysmal sites or venous territories); (ii) extra-parenchymal haemorrhagic findings, referring to haemorrhage extending beyond the brain parenchyma into intraventricular, subarachnoid or subdural spaces; (iii) non-haemorrhagic abnormalities (eg, spontaneous hypodensity of venous structures, and concomitant ischaemic lesions) and (iv) absence of CT markers of SVD.

While the NCCT findings identified in this review may raise suspicion for secondary causes of ICH, they are generally insufficient to confirm or, more critically, to exclude a secondary aetiology. Current aetiological classification systems, such as classification of ICH (CLAS-ICH) and classification of cerebral haemorrhage (CADMUS), rely heavily on vascular imaging and brain MRI to differentiate SVD from secondary causes.^3^^,^^39^ As such, NCCT features should be considered tools for risk stratification, helping to prioritise patients for further diagnostic workup.

Several clinical-imaging scores integrating NCCT have been proposed to guide selection for vascular imaging in the acute setting. These include the DIAGRAM score^40^ (which incorporates younger age, lobar or posterior fossa ICH location and absence of SVD signs), the simple clinical score^41^ (which factors in young age, hypertension, lobar or posterior fossa ICH location, IVH and oral anticoagulant use) and the secondary ICH score^25^ (which includes clinical variables such as history of hypertension or impaired coagulation, sex, age and NCCT features such as ICH location, calcifications, enlarged vessel and spontaneous hyperdensity of venous structures). Reported c-statistics for these scores in predicting an underlying macrovascular cause were 0.66, 0.70 and 0.87, respectively. Our comprehensive synthesis of NCCT findings may offer a foundation for developing improved risk scores, ideally integrating clinical and imaging data, to more accurately predict a broader spectrum of secondary ICH aetiologies.

Accurate and timely identification of the underlying cause of ICH has important clinical implications. Specific treatments vary depending on the aetiology. For example, endovascular intervention may be indicated to prevent rebleeding in patients with macrovascular lesions; corticosteroids can reduce mass effect in tumour-associated haemorrhage; antibiotics are critical for infectious causes; urgent anticoagulation is essential for CVT; delayed prophylactic anticoagulation may be appropriate in cases related to melanoma metastases or coagulopathy and early secondary stroke prevention with anticoagulation is warranted for haemorrhagic transformation of cardioembolic infarction. These examples underscore the need for early aetiologic differentiation, as management strategies can differ markedly and may carry significant risks if misapplied.^42^

In emergency settings, particularly in stroke centers where routine CTA is not performed during the hyperacute care of ICH management, NCCT findings suggestive of secondary ICH may be especially valuable. This is even more relevant in low- and middle-income countries, which carry the highest global burden of ICH but often lack access to more advanced imaging modalities such as CTA or MRI.^2^ Moreover, it might also be valuable for patients who cannot undergo MRI or have a contraindication to CT contrast administration (eg, due to allergy or renal impairment). In these contexts, risk stratification based on NCCT findings may be critical to guide appropriate and timely further diagnostic work-up. Despite this potential, we identified a notable gap in the literature: few studies have been specifically designed to diagnose, prognosticate or guide treatment of secondary ICH based on NCCT findings. Some of the evidence included in this review was derived from expert reviews rather than primary studies, underscoring the need for more direct, high-quality investigations. To date, most neuroimaging research in acute ICH has focused on predicting haematoma expansion, with limited emphasis on aetiological classification.^43^ Future prospective clinical studies are needed to validate the diagnostic utility of specific NCCT findings in patients with acute ICH who undergo a comprehensive aetiological workup.

## Conclusion

We identified a range of NCCT findings that may raise early suspicion for a secondary aetiology of ICH. Recognising these patterns can help stratify patients who may benefit from further vascular or advanced neuroimaging, particularly in resource-limited settings where access to such imaging is constrained. Prompt identification of a secondary ICH cause has important clinical implications, enabling timely, aetiology-specific interventions that can improve patient outcomes.

## Supplementary Material

aakaf010_Supplementary_Figure_1_NCCT_findings_suggestive_of_secondary_ICH_ESJ_R1

## Data Availability

The corresponding author will provide data of the study upon reasonable request.
