# Supplementary material for: Non-contrast CT findings suggestive of secondary intracerebral haemorrhage
Source: Eur Stroke J. 2026 Jan 1;11(1):aakaf010. doi: 10.1093/esj/aakaf010 (PMC12866644; doi:10.1093/esj/aakaf010)

**Supplementary Figure 1. Example of a case with coexisting hemorrhagic and ischemic lesions**(A) Non-contrast CT show a left fronto-insular-temporal intraparenchymal hemorrhage with concomitant right insular hypodense suggestive of recent ischemic lesion (white arrow). (B) sagittal and (C) coronal view of CT angiography show an infective aneurysm (yellow arrows). The presence of coexisting ischemic and hemorrhagic lesions and an infective aneurysm is suggestive of an underlying endocarditis etiology.


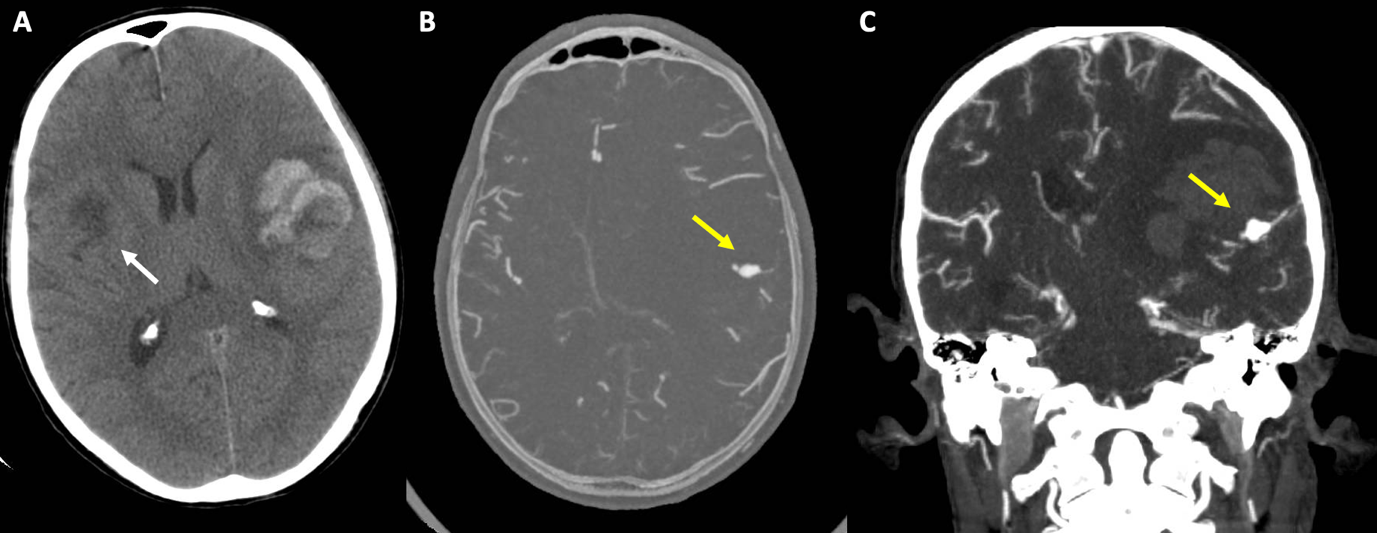

Supplement: aakaf010_Supplementary_Figure_1_NCCT_findings_suggestive_of_secondary_ICH_ESJ_R1 [file aakaf010_supplementary_figure_1_ncct_findings_suggestive_of_secondary_ich_esj_r1.docx]
